# Neurological Symptoms of COVID-19: The Zonulin Hypothesis

**DOI:** 10.3389/fimmu.2021.665300

**Published:** 2021-04-26

**Authors:** Sílvia Llorens, Eduardo Nava, Mónica Muñoz-López, Álvaro Sánchez-Larsen, Tomás Segura

**Affiliations:** ^1^ Department of Medical Sciences, Faculty of Medicine of Albacete, University of Castilla-La Mancha, Albacete, Spain; ^2^ Centro Regional de Investigaciones Biomédicas (CRIB), University of Castilla-La Mancha, Albacete, Spain; ^3^ Servicio de Neurología, Hospital General Universitario de Albacete, Albacete, Spain; ^4^ Instituto de Investigación en Discapacidades Neurológicas (IDINE), University of Castilla-La Mancha, Albacete, Spain

**Keywords:** zonulin, Toll-like receptor4, tight junctions, blood-brain barrier, neurological symptoms, gastrointestinal symptoms, haematogenous route, SARS-CoV-2

## Abstract

The irruption of SARS-CoV-2 during 2020 has been of pandemic proportions due to its rapid spread and virulence. COVID-19 patients experience respiratory, digestive and neurological symptoms. Distinctive symptom as anosmia, suggests a potential neurotropism of this virus. Amongst the several pathways of entry to the nervous system, we propose an alternative pathway from the infection of the gut, involving Toll-like receptor 4 (TLR4), zonulin, protease-activated receptor 2 (PAR2) and zonulin brain receptor. Possible use of zonulin antagonists could be investigated to attenuate neurological manifestations caused by SARS-CoV-19 infection.

## Introduction

SARS-CoV-2 emerged in December 2019 and rapidly caused a global pandemic extending along 2020. Since March 2020, when the World Health Organization (WHO) declared the pandemic nature of the problem, the virus has caused 102 million infections and approximately 2 million deaths (https://www.worldometers.info). SARS-CoV-2 causes both upper and lower respiratory tract infections (1). The infection is characterized by a process ranging from asymptomatic and mild disease to severe systemic symptoms involving mainly the lung and gastrointestinal (GI) tract, and finally, it can cause multi-organ failure (2). Transmission of SARS-CoV-2 is mainly caused by human respiratory droplets or aerosol carrying the virus, which enters the airways of the host and infects epithelial cells (3). One of the factors determining severity of COVID-19 is the aggressive inflammatory response from the host, which can cause severe systemic damage by the so-called “cytokine storm” (4). It is well known that SARS-CoV-2 infects the host cell by binding its spike protein to the receptor binding domain of angiotensin-converting enzyme 2 (ACE2) (5).

## Neurological Manifestations of COVID-19

Acute hypoxia and acute respiratory disease syndrome (ARDS) are two of the major causes of the high fatality rate of COVID-19 patients (6). However, this virus frequently causes neurological manifestations (such as headache, dizziness, impaired consciousness, stroke, encephalitis, anosmia, dysgeusia, Guillain-Barre syndrome, ataxia, etc.) (7, 8). The percentage of COVID-19 patients that develop some form of neurologic symptom varies depending on the studies. Most researchers accept that it is around or above 50% of the patients. In fact, the study by Romero-Sánchez et al., found that 57.4% of hospitalized COVID-19 patients exhibited some type of neurological manifestation (8). These figures are important because COVID-19 patients with neurological disorders have an increased risk of in-hospital mortality and lower rates of discharge home compared to COVID-19 patients without neurological disorders.

Possible routes of entry of this virus into the central nervous system (CNS) have been recently reviewed by Kumar et al. (9). Distinctive symptoms of the infection by this virus, such as anosmia, indicate a potential viral neurotropism, and a direct route of entry into the CNS *via* the olfactory nerves (9), which may lead to viral replication and CNS invasion (10). Other mechanisms of brain invasion that have been postulated are a retrograde spread through the vagus nerve and a hematogenous route (9). There is evidence that neurological damage in COVID-19 patients is not primarily due to direct invasion of the virus into the CNS. For example, in the majority of infected patients with severe neurological manifestations, in whom real-time RT-PCR was performed, the cerebrospinal fluid (CSF) was positive for SARS-CoV-2 in less than 3% of the patients (11).

## Neurological Versus Gastrointestinal COVID-19

SARS-CoV-2 infected patients also experience enteric symptoms (such as fever, myalgia, lethargy, dry cough, dyspnea, anorexia, abdominal pain, and diarrhea) (12). While gastrointestinal symptomatology is, behind the respiratory, among the most frequent in COVID-19 patients (13, 14), whether digestive and neurological disorders combine in a significant manner is unclear. We collected the epidemiological data and clinical symptoms of every COVID-19 patient (945 patients) admitted to our hospital (University General Hospital of Albacete), during March 2020. We found that neurological and gastrointestinal symptoms (unrelated to the prescribed drugs) were frequent in hospitalized patients: 54.5% and 53.2%, respectively. Interestingly, we found that suffering from gastrointestinal symptoms was significantly associated with the display of some neurological symptom (p = 0.027). Therefore, in these hospitalized patients, gastrointestinal symptoms are a risk factor for developing mild neurological complications such as headache, myalgia, anosmia, or dysgeusia. **Table 1** displays the details of the different data.

**Table 1:** Relationship of neurological complications and gastrointestinal symptoms during the course of coronavirus disease 2019.

		Gastrointestinal symptoms		*p* Value
	Total patients (n = 945)	No (n = 442)	Yes (n = 503)	
Any neurological symptom	No (n = 430)	218	212	0.027
	Yes (n = 515)	224	291	
Myalgias	No (n = 781)	389	392	< 0.001
	Yes (n = 164)	53	111	
Headache	No (n = 805)	391	414	0.008
	Yes (n = 140)	51	89	
Anosmia	No (n = 896)	429	467	0.004
	Yes (n = 49)	13	36	
Dysgeusia	No (n = 883)	425	458	0.002
	Yes (n = 62)	17	45	
Psychiatric symptoms	No (n = 768)	368	400	0.142
	Yes (n = 177)	74	103	
Meningoencephalitis	No (n = 943)	441	502	1.000
	Yes (n = 2)	1	1	
Demyelinating diseases	No (n = 944)	441	503	0.486
	Yes (n = 1)	1	0	
Seizures	No (n = 935)	433	502	0.008
	Yes (n = 10)	9	1	
Movement disorders	No (n = 939)	437	502	0.104
	Yes (n = 6)	5	1	
Guillain-Barre syndrome	No (n = 944)	441	503	0.486
	Yes (n = 1)	1	0	
Ischemic stroke	No (n = 930)	428	502	0.001
	Yes (n = 15)	14	1	
Brain hemorrhage	No (n = 940)	438	502	0.135
	Yes (n = 5)	4	1	

Data were collected and analyzed using SPSS version 25 software (SPSS, Chicago, IL, USA). The ratios were compared using the χ2 test, and the Fisher exact test when the sample size was too small, considering a value p less than 0.05 as statistically significant.

## Blood-Brain Barrier Disruption

If we assume that the neurological involvement in COVID-19 disease is not mainly due to direct invasion by the virus, why does it occur? Most authors currently argue that it is caused by disruption of the blood-brain barrier (BBB), (15).Even though the respiratory tract is the main site of infection and viral replication, growing evidence indicates an extrapulmonary dissemination of the virus (16) (17) (18). A paracellular pathway, disrupting epithelial or endothelial barriers is an alternative route that viruses use to enter the bloodstream and thereby contribute to viral dissemination. Viruses *via* this pathway disrupt epithelial or endothelial barriers including the BBB. To achieve this, tight junctions (TJ) must be disassembled. TJ are complex and dynamic structures involved in several key functions of epithelial and endothelial barriers under physiological conditions as well as in pathological circumstances (19). TJ impairment specifically occurs in severe COVID-19 patients (20).

## Zonulin

Zonulin is a 47 KDa protein initially described by Wang et al. in 2000 (21) that works as an endogenous regulator of intestinal paracellular permeability disassembling tight junctions (TJ). Zonulin has been mainly localized in the GI tract (22) and linked to GI disease like coeliac disease (23). Zonulin has been reported to be upregulated also in extraintestinal tissues such as the lung (24) and brain tissue (25). Concerning the lung, Rittirsch et al. showed that the zonulin peptide antagonist, AT-1001, attenuated acute lung injury in mice (24). Concerning the brain, zonulin receptor has been found in the brain tissue (26). Skardelly et al. demonstrated that zonulin is able to reach the brain and increase BBB permeability (25).

## Hypothesis

The aim of the present review is to develop a feasible hypothesis complementary to the current information that exists regarding the SARS-CoV-2 virus’ journey to the brain (9). From our results, the core of our hypothesis emerges to provide a mechanism for both intestinal and cerebral zonulin expression linked to SARS-CoV-2 infection. Furthermore, to label zonulin as one of the factors responsible for destroying or disrupting the BBB is crucial in order to find a possible therapy directed against the neurological manifestations that occur in COVID-19 patients. In fact, the already mentioned zonulin peptide antagonist, AT-1001, has been proposed as a specific anti-SARS-CoV-2 drug (27).

**Figure 1:**
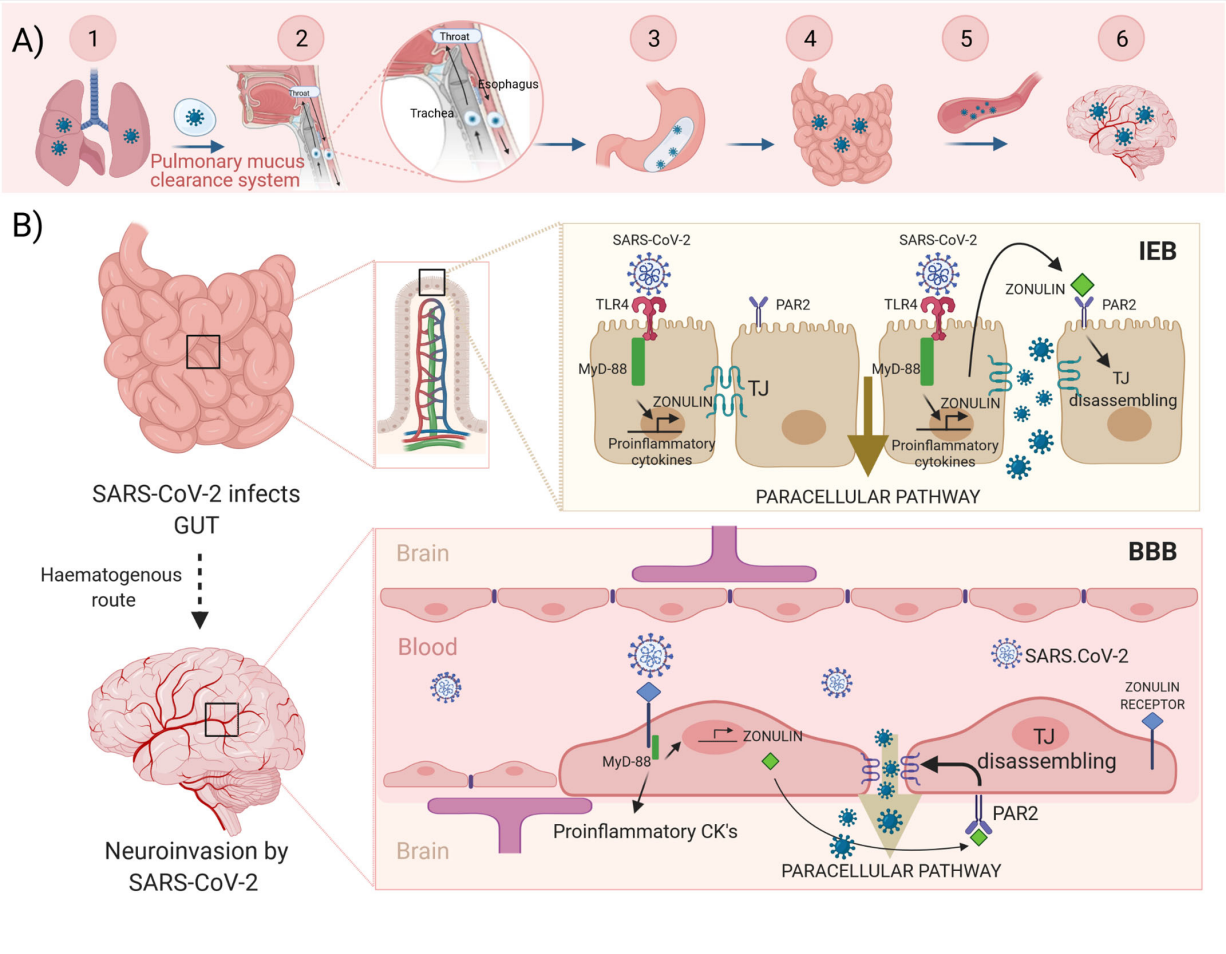
Hypothesis illustrated. Upper panel **(A)** Scheme of the SARS-CoV-2 journey towards to the brain: 1.-Infected lungs. 2.- The pulmonary mucus clearance system. 3.- Enteroinvasion. 4.- An increased intestinal permeability allows the virus to access the bloodstream and spread. 5.- Haematogenous route. 6.- Neuroinvasion. Lower panel **(B)** SARS-CoV-2 virus reaches the intestine protected by the mucus from infected lungs. The virus can bind and activate the TLR4, which via MyD-88 could activate both zonulin and proinflammatory cytokines expression. Zonulin, secreted to the lumen, binds with PAR2 inducing disassembling of TJ. Now, the virus can use the paracellular pathway to access the circulatory system and reach the brain. Zonulin can also access to the bloodstream. Then, the virus, via brain zonulin receptor, can induce expression of zonulin and consequently increase permeability of the BBB by inducing disassembling of TJ. This causes neuroinvasion of the virus. TLR4, toll-like receptor 4; MyD-88, myeloid differentiation primary response gene 88; PAR2, protease-activated receptor 2; TJ, tight junctions; IEB, intestinal epithelial barrier; BBB, blood-brain barrier. Created with BioRender.com.

Our hypothesis is illustrated in the **Figure 1**: SARS-CoV-2 reaches the intestine protected by the mucus from infected lungs by the pulmonary mucus clearance system. Viruses land on the mucus layer and are moved by cilia up the trachea, through the vocal chords, and then swallowed and cleared by the gastrointestinal tract. During a cough, central airways narrow, and globs of mucus are propelled forcefully by a column of air moving at high velocity directly into the pharynx where they mix with saliva from the mouth and are swallowed into the esophagus (28). Then, the virus can bind enterocyte surface ACE2 and replicates. However, it can also bind and activate the TLR4 receptors, which *via* MyD-88 could activate zonulin and promote proinflammatory cytokine overexpression. Zonulin, secreted to the lumen, binds PAR2 inducing the TJ disassembly of intestinal epithelial barrier (IEB). At this point, the virus can use the paracellular pathway to access the circulatory system and reach the brain. Then, the virus, *via* brain zonulin receptor, can induce the overexpression of zonulin, which increases BBB permeability through a similar TJ disassembly mechanism finally causing neuroinvasion. On the other hand, both the complement system activated by zonulin (see below) and the cytokine storm inducted during viral infection might potentiate the disruption of the BBB and account for the neurological symptoms exhibited by COVID-19 patients.

We have analyzed data from previous studies and detailed in the following paragraphs, the scientific evidence that support our hypotheses:

### SARS‐CoV‐2 Directly Infects and Replicates in Intestinal Cells

Digestive symptoms as nausea, vomiting, diarrhea, abdominal pain, and hematochezia, are frequently found in COVID-19 patients (13, 14). In addition to the lungs, several lines of evidence point to the gut as a possible target of SARS-CoV-2. Hoffmann et al., showed that SARS-CoV-2 employs ACE2 as a cellular receptor and that its spread is dependent on the activity of transmembrane serine protease 2 (TMPRSS2) (29). Later, Kumar and colleagues found enriched expression of ACE2 and significantly enhanced expression of TMPRSS2 in intestinal epithelial cells (30).

The following data provides the basis to suggest that SARS‐CoV‐2 may be capable of infecting and replicating in the intestine:

-ACE2, the key host cell entry receptor for SARS‐CoV‐2, is highly expressed in the brush border of the intestinal epithelial cells (31).
-High levels of expression of ACE2, TMPRSS2 and TMPRSS4, which are essential factors for host cell infection by SARS-CoV-2, have been observed in lower GI epithelial cells (32).
-The presence of SARS‐CoV‐2 viral RNA in the stool samples from infected patients that persists over 1-5 weeks has been reported even with serum specimens negative for the presence of the virus (33, 34).
-SARS-CoV-2 RNA detection and intracellular staining of viral nucleocapsid protein has been reported in gastric, duodenal, and rectal epithelia (18).
-The hemorrhagic colitis reported during SARS-CoV-2 infection involves the GI tract in the transmission of SARS-CoV-2 infection (35).
-Typical coronavirus virions have been observed by electron microscopy in rectal biopsy samples (36–38).
-COVID‐19 patients with concomitant GI symptoms show poorer clinical outcomes requiring more often mechanical ventilation (39).
-Infection of mature human enterocytes by SARS-CoV-2, through viral fusion and entry *via* ACE2, has been demonstrated *in vitro* in studies based on monolayer cultures of intestinal epithelial cells (40, 41). This has also been shown in human small intestinal organoids (which are the 3D structures that are grown from adult stem cells) (42, 43). Direct viral infection and replication of SARS-CoV-2 in the intestinal epithelium and endothelium has been demonstrated by means of organ-on-a-chip technology (44).

### Access of SARS-CoV-2 to the Small Bowel

We hypothesize that the intestine is an entry site for SARS-CoV-2 towards the CNS. However, this must involve a reduced gastric acid level because the virus cannot survive a normal gastric acid environment of pH 1.5-3. There is evidence that SARS-CoV-2 can survive a pH level above 3 (45). Thus, in a hypochlorhydria the virus can survive the route from the mouth to the bowel, where the virus could directly infect enterocytes (46). Hirose et al, have reported that viscous sputum or nasal discharge may protect human influenza A and B virions (IAV/IBV). These are viruses that are inactivated by low pH and vulnerable to surfactants such as bile (47). Usually, the mucus secreted by cells lining the intestinal tract serves to clear respiratory viruses, including IAV/IBV. Therefore, the presence of the SARS-CoV-2 virus in the gut may be due to the self-ingestion of mucus from the airways by coughing discharge. Viral particles would preserve their infectivity protected by mucus from the degrading action of gastric acid, bile and pancreatic juices. Once SARS-CoV-2 has overcome these defensive barriers, it penetrates into host cells through viral fusion *via* ACE2 accomplished by the mentioned serine proteases TMPRSS2 and TMPRSS4 (30). Upon viral entry, virus-specific RNA and proteins are synthesized in the cytoplasm to assemble new virions (48), which can be released to the GI tract. This would explain the presence of viral RNA in stool samples from COVID-19 patients. However, it should not be forgotten that viruses and bacteria frequently use other pathways to disseminate, such as the paracellular pathway. We postulate that the SARS-CoV-2 virus entering *via* the paracellular pathway is a contributing factor in the exacerbated immune response of the host’s intestine, culminating with the cytokine storm and, finally leading to the dissemination of the virus to the brain.

### Association of SARS-CoV-2 and TLR4

In addition to the ACE2, binding to toll-like receptors (TLRs), especially TLR-4, may contribute to the infectivity and pathogenesis of SARS-CoV-2 (49). TLR4 is not only present in immune cells, but is also expressed in intestinal epithelial cells (50). TLR4 is overexpressed in chronic inflammatory conditions and participates in the antiviral defense against RNA viruses such as respiratory syncytial virus (51) or coxsackievirus B4 (52). Also, TLR4 is known to recognize the envelope (Env) proteins of mouse mammary tumor virus and murine leukemia virus (53). Recognition of viral particles produces activation and dimerization of TLR4 and triggers two distinct signal transduction pathways mediated by the adaptor proteins: Toll/IL-1 receptor (TIR) domain-containing adaptor inducing IFN-β (TRIF) and MyD88. TRIF activates a signal transduction pathway responsible for the production of additional type I interferons, which are involved in antimicrobial host defense. MyD88, which has been reported to be involved in the release of zonulin (54), activates a signal transduction pathway resulting in the release of the NF-κB, a transcription factor required to induce the gene expression of most proinflammatory cytokines, like IL-1, IL-6 or TNF-α (50).

Existing data supports the association of COVID-19 with TLR4:

-The main cytokines involved in severe COVID-19 cases (IL-6 and TNF-α) are downstream of the TLR4 signaling pathway (55).
-An *in-silico* study demonstrated that the spike protein of SARS‐CoV‐2 binds with surface TLRs (TLR1, 4, and 6), the binding being especially strong with TLR4 (56).
-A ligand of TLR4 was the most highly increased among other inflammatory mediators in COVID 19 severe patients when compared to healthy control (57).

We postulate that the virus’ spike protein activates TLR4, triggers MyD88 signal transduction pathways that would end in the overexpression of zonulin. Once secreted from enterocytes, zonulin binds specific receptors leading to the phosphorylation of TJ proteins and produces an increase in intestinal permeability and, consequently, the disassembly of these proteins.

### Activation of TLR4 by SARS-CoV-2 Would Induce the Expression of Zonulin

Zonulin signaling has been postulated to be dependent upon protease-activated receptor 2 (PAR2) (58). PAR2 mRNA is strongly expressed in the small intestine, colon, liver, and pancreas. Immunoreactive PAR2 is localized to the apical and basolateral membrane of enterocytes and numerous other tissues (59). It has been also reported that PAR2 plays a key role in modulating several diseases such as experimental autoimmune encephalomyelitis, multiple sclerosis (MS) (60) and arthritis (61). This signaling protein interacts physically and functionally with TLR4 (62). Several studies have investigated the possible connection between PAR2 and TLR4-mediated signaling pathways. For example, concurrent activation of PAR2 and TLR4 by the PAR2 activating protein (AP) and LPS, respectively, amplifies NF-κB activation and IL-6 production in endothelial cells (63). Furthermore, PAR2 expression is upregulated by pro-inflammatory cytokines such as TNFα and IL-1β, leading to a self-sustaining and amplified inflammatory process (64).

It is well established that IL-6 is the proinflammatory cytokine that is found in highest levels in serum of severe COVID-19 patients (65). Indeed, higher than normal IL-6 levels are detected in COVID-19 patients requiring hospitalization or with acute respiratory failure (65, 66). It is interesting that the promoter of zonulin is under IL-6 control (67). Thus, overexpression of zonulin may be related to expression of IL-6 by MyD88.

### Zonulin Activates the Complement System

Activation of the complement system is the first response of the host innate immune system against any foreign invasion, like SARS‐CoV‐2 infection. However, uncontrolled complement activation can be damaging. This possibly initiates the clinical complications affecting others organs in COVID-19 patients (68). Zonulin, both *in vitro* and *in vivo*, induces activation of components of complement C3 and C5 facilitating acute lung injury (ALI) by an increased accumulation of neutrophils and cytokines (24). With regard to this, it is worth noting that during SARS‐CoV-2 infection, the activation of complement component C3 exacerbates ARDS and deposits of C3‐C5 complements are abundant in lung biopsies from COVID‐19 patients (69).

### Neuroinvasion by SARS-CoV-2 From Enteroinvasion

Two main pathways have been proposed for the entry of neurotropic respiratory viruses into the CNS: a retrograde neuronal route and a hematogenous route. In the retrograde neuronal route, viruses undergo retrograde axonal transport to reach the neuron cell bodies in the peripheral and or CNS. In the hematogenous route, viruses gain access by infecting endothelial cells of the BBB, epithelial cells of the blood-cerebrospinal fluid barrier of the choroid plexus, or alternatively use inflammatory cells as Trojan horses to gain access into the CNS (70).

Recently, it has been postulated that SARS‐CoV‐2 neuroinvasion may occur *via* the vagal afferents from the GI, highlighting a role for gut-brain axis in the pathogenesis of the disease (71). The gut-brain axis has been involved in the pathogenesis of neuroinflammatory diseases such as MS, epilepsy, and stroke (72). The enteric nervous system is strongly interconnected with enteric glial cells, which express the major histocompatibility complex class II and therefore acts as antigen‐presenting cells for immune cells of the gut‐associated lymphoid tissue (GALT). Upon activation by viral infection, GALT initiates immune responses, increases in the endothelium permeability and release of higher IL‐6 levels and other inflammatory mediators. It contributes to ARDS as observed in the COVID‐19‐induced cytokine storm (71, 73). Esposito et al., suggest that SARS‐CoV‐2–related diarrhea and the GI dysfunction serve as a possible marker of the involvement of the enteric nervous system/enteric glial cell in the pathogenesis of GI COVID-19 (71). Additionally, Kumar and coworkers proposed intestinal ACE2 as an essential entry factor involved in the digestive symptoms of COVID-19 (30). Thus, the gut could be used as a gateway through which viruses can either directly neuroinvade or indirectly immunologically prepare the enteric nervous system to achieve an ascending route towards the CNS through intestinal vagal afferents.

SARS‐CoV‐2 has been shown to directly infect engineered human blood vessel organoids *in vitro*. Furthermore, viral-like particles have been observed in brain capillary endothelium actively crossing the endothelial cells (74, 75). This suggests that the hematogenous route is the most likely route for SARS-CoV-2 to the brain. Considering all this data together and in line with our results (**Table 1**), we propose that the increased intestinal permeability caused by overexpression of zonulin opens an entry door for SARS‐CoV‐2. Through this way, the virus reaches the bloodstream or the lymphatic system, infects endothelial cells of blood or lymphatic vessels, infects local tissues, and then is disseminated to many organs, including the CNS.

### SARS-CoV-2, Zonulin and BBB. Integrating Concepts

Increasing the permeability of the BBB is a common mechanism of damage used by numerous viruses (76, 77). The IEB and the BBB are formed by epithelial and endothelial cells, respectively. Both barriers exhibit similarities. Both are regulated by interactions with glial cells that are connected with the enteric nervous system and the central nervous system (CNS), their cells are sealed by tight junctions and are sensitive to disruption by external stimuli (78). Breakdown of the BBB plays an important role in the pathogenesis of numerous brain diseases, including neurological diseases such as stroke, epilepsy, and MS, brain infarction, or brain hemorrhage (79). BBB disruption significantly contributes to brain inflammation through the leakage of plasma factors into the brain, blocking of endothelial pericyte interaction, activation of glial cells, and induction of immune cell migration into brain tissue. On the other hand, brain inflammation facilitates BBB disruption through digestion of the basement membrane by proteinases and injury of BBB cells (80).

As mentioned above, COVID-19 patients with neurological disorders have increased risk of in-hospital mortality and lower rates of discharge home compared to COVID-19 patients without neurological disorders (7).

Other types of receptors or cellular entry mode for SARS‐CoV‐2 have been considered in the nervous cells or tissues (81). Using a combination of structural and molecular approaches, Fantini and co-workers demonstrated that the ganglioside‐binding domain (111–158) at the tip of the N‐terminal domain of the spike protein of SARS‐CoV‐2, as well as sialic acids linked to glycoproteins of host cell surface can also serve as an additional cellular entry for SARS‐CoV‐2 (82).

Several clues point to the possibility that zonulin is implicated in the neurological manifestations of SARS-CoV-2 infection: zonulin could act in the brain throughout the BBB disruption. Support for this possibility arises from a recent report using the zonulin agonist peptide AT-1002 which shows that zonulin is associated with an increased permeability of the BBB (83). Over-activation of the complement system has also been linked to an enhanced permeability of this barrier. This is the case of neuroinflammatory diseases involving C5 signaling through its G‐protein coupled receptor (84). Besides, and as stated above, zonulin can induce the activation of components C3 and C5 of complement system (24). TLR4 and PAR2, necessary for the overexpression and functional of zonulin, are expressed in neuronal and glial cells and are involved in development and progression of neurodegenerative diseases with an inflammatory component (85). Moreover, the human brain receptor for zonulin is a glycoprotein that contains multiple sialic acid residues (26), which, interestingly, is the new receptor postulated above for SARS-CoV-2. A remarkable *in vitro* study by Karyekar et al., studying a zonulin analogue (named Zot, a choleric toxin), suggested that its receptor might also be expressed in the endothelium of brain capillaries, and that it could dissemble TJs *in vivo* (86). Taking altogether, these evidences introduce in our hypothesis, a possible mechanism responsible for the neurological symptomatology caused by the virus: upon arrival of the virus to the brain *via* the hematogenous route, this would bind to the zonulin receptor and thereby, activate the expression of zonulin *via* MyD88. Zonulin would be secreted to the brain tissue side, bind to its receptor (PAR2), induce the disassembly of TJs, disrupt the BBB and consequently allowing the entry of the virus to the brain. On the other hand, during SARS-CoV-2 infection the overexpression of zonulin in the brain could also be dependent on the disruption of the IEB, resulting in enhanced secretion of cytokines into the bloodstream that can reach the brain capillaries. Although, under physiological conditions, most cytokines exert their effects locally at secretion sites, under pathological conditions high levels of them are secreted into the bloodstream, acting on distal cells in an endocrine manner to mediate systemic responses (87). Since high levels of the cytokine IL-6 are found in COVID-19 patients (65) and the promotor of zonulin is under control of this cytokine (67), the overexpression of zonulin in the brain could also be related to the presence of IL-6 in the brain capillaries.

## Testing the Hypothesis

Further *in situ* or *in vivo* studies will be necessary to establish a substantial role of zonulin in COVID-19 neuropathology. In this regard, several unresolved questions in this review must be tested, as following:

Crystal structure studies to confirm the interaction between SARS-CoV-2 spike protein and human TLR4 need to be performed (56).

A biochemical and molecular characterization of the brain zonulin receptor as well as a detail localization in the brain of the zonulin receptor needs to be established (26). On the other hand, the confirmation of the existence of the zonulin receptor in the luminal side of the capillaries constituting the BBB, is also necessary. It would also be necessary to verify that intestinal zonulin reaching the brain *via* the bloodstream could bind to the capillary zonulin receptor. In this sense, a biochemical and molecular study of intestinal and brain zonulin to test their differences would also be of great interest.

A longitudinal dataset related to the mechanism of pathogenesis of SARS-CoV-2 involving TJ impairment must be performed (20).

In summary, we hypothesize that after lung infection, SARS-CoV-2, protected with airway-borne mucus, reaches the gut undigested. Once here, the spike protein binds TLR4 and, *via* MyD88, induces the expression of proinflammatory cytokines, especially IL-6. This cytokine promotes the overexpression of zonulin, which, *via* PAR2, disassembles TJ, opening paracellular pathways and allowing the virus to pass through. The virus can now infect vascular endothelial cells and disseminate to the CNS through a hematogenous route. Once at the BBB, SARS-CoV-2 binds zonulin receptor and promotes zonulin release. Then zonulin, *via* PAR2, induces the BBB disruption allowing the virus to enter.

We hope that this article will open up the possibility of investigating the effect of zonulin antagonists on the attenuation of neurological symptoms caused by SARS-CoV-19 infection.

## Limitations

Our data were obtained retrospectively, so selection bias may arise and some important information could be missing. Finally, this study is hospital-based, so it does not necessarily reflect the incidence of gastrointestinal or neurologic symptoms of patients with mild COVID-19.

## Data Availability Statement

The datasets presented in this study can be found in online repositories. The names of the repository/repositories and accession number(s) can be found below: Raw data were generated at University General Hospital Universitario from Albacete. Study design and data collection regarding ALBACOVID registry are described in (8).

## Ethics Statement

The studies involving human participants were reviewed and approved by Comité de Ética en Investigación Clínica (University of Castilla-La Mancha) identifier: 2020/04/043. The patients/participants provided their written informed consent to participate in this study.

## Author Contributions

Conceptualization, SLL. Investigation, SLL, ÁS-L and TS. Resources, SLL, EN, MM-L, ÁS-L and TS. Writing—original draft preparation, SLL. Writing—review and editing, EN, MM-L, ÁS-L and TS. Visualization, SLL. Supervision, SLL, EN, MM-L, ÁS-L and TS. All authors contributed to the article and approved the submitted version.

## Conflict of Interest

The authors declare that the research was conducted in the absence of any commercial or financial relationships that could be construed as a potential conflict of interest.

## References

[1] Wölfel R Corman VM Guggemos W Seilmaier M Zange S Müller MA. Virological Assessment of Hospitalized Patients With Covid-2019. Nature (2020) 581(7809):465–9. 10.1038/s41586-020-2196-x 32235945

[2] Wiersinga WJ Rhodes A Cheng AC Peacock SJ Prescott HC. Pathophysiology, Transmission, Diagnosis, and Treatment of Coronavirus Disease 2019 (Covid-19): A Review. JAMA (2020) 324(8):782–93. 10.1001/jama.2020.12839 32648899

[3] Zhang R Li Y Zhang AL Wang Y Molina MJ. Identifying Airborne Transmission as the Dominant Route for the Spread of COVID-19. Proc Natl Acad Sci U S A (2020) 117(26):14857–63. 10.1073/pnas.2009637117 PMC7334447 32527856

[4] Wong CK Lam CW Wu AK Ip WK Lee NL Chan IH. Plasma Inflammatory Cytokines and Chemokines in Severe Acute Respiratory Syndrome. Clin Exp Immunol (2004) 136(1):95–103. 10.1111/j.1365-2249.2004.02415.x 15030519 PMC1808997

[5] Li W Greenough TC Moore MJ Vasilieva N Somasundaran M Sullivan JL. Efficient Replication of Severe Acute Respiratory Syndrome Coronavirus in Mouse Cells is Limited by Murine Angiotensin-Converting Enzyme 2. J Virol (2004) 78(20):11429–33. 10.1128/JVI.78.20.11429-11433 PMC521845 15452268

[6] Goh KJ Choong MC Cheong EH Kalimuddin S Duu Wen S Phua GC. Rapid Progression to Acute Respiratory Distress Syndrome: Review of Current Understanding of Critical Illness From Covid-19 Infection. Ann Acad Med Singap (2020) 49(3):108–18. 10.47102/annals-acadmedsg.202057 32200400

[7] Losy J. Sars-Cov-2 Infection: Symptoms of the Nervous System and Implications for Therapy in Neurological Disorders. Neurol Ther (2020) 23:1–12. 10.1007/s40120-020-00225-0 PMC7681771 33226565

[8] Romero-Sánchez CM Díaz-Maroto I Fernández-Díaz E Sánchez-Larsen Á Layos-Romero A García-García J. Neurologic Manifestations in Hospitalized Patients With Covid-19: The Albacovid Registry. Neurology (2020) 95(8):e1060–70. 10.1212/WNL.0000000000009937 PMC7668545 32482845

[9] Kumar A Pareek V Prasoon P Faiq MA Kumar P Kumari C. Possible Routes of SARS-Cov-2 Invasion in Brain: in Context of Neurological Symptoms in COVID-19 Patients. J Neurosci Res (2020) 98(12):2376–83. 10.1002/jnr.24717 32869376

[10] Yan CH Faraji F Prajapati DP Boone CE DeConde AS. Association of Chemosensory Dysfunction and COVID-19 in Patients Presenting With Influenza-Like Symptoms. Int Forum Allergy Rhinol (2020) 10(7):806–13. 10.1002/alr.22579 PMC7262089 32279441

[11] Pezzini A Padovani A. Lifting the Mask on Neurological Manifestations of COVID-19. Nat Rev Neurol (2020) 16(11):636–44. 10.1038/s41582-020-0398-3 PMC7444680 32839585

[12] Zhu N Zhang D Wang W Li X Yang B Song J. A Novel Coronavirus From Patients With Pneumonia in China, 2019. N Engl J Med (2020) 382(8):727–33. 10.1056/NEJMoa2001017 PMC7092803 31978945

[13] Song Y Liu P Shi XL Chu YL Zhang J Xia J. Sars-Cov-2 Induced Diarrhoea as Onset Symptom in Patient With Covid-19. Gut (2020) 69(6):1143–4. 10.1136/gutjnl-2020-320891 32139552

[14] Wan J Wang X Su S Zhang Y Jin Y Shi Y. Digestive Symptoms and Liver Injury in Patients With Coronavirus Disease 2019 (Covid-19): A Systematic Review With Meta-Analysis. JGH Open (2020) 4(6):1047–58. 10.1002/jgh3.12428 PMC7731824 33319036

[15] Hernández-Fernández F Sandoval Valencia H Barbella-Aponte RA Collado-Jiménez R Ayo-Martín Ó Barrena C. Cerebrovascular Disease in Patients With COVID-19: Neuroimaging, Histological and Clinical Description. Brain (2020) 143(10):3089–103. 10.1093/brain/awaa239 PMC7454411 32645151

[16] Chen Z Li G. Immune Response and Blood–Brain Barrier Dysfunction During Viral Neuroinvasion. Innate Immun (2020) 27(2):110–7. 10.1177/1753425920954281 PMC7882805 32903111

[17] Pennisi M Lanza G Falzone L Fisicaro F Ferri R Bella R. Sars-Cov-2 and the Nervous System: From Clinical Features to Molecular Mechanisms. Int J Mol Sci (2020) 21(15):5475. 10.3390/ijms21155475 PMC7432482 32751841

[18] Xiao F Tang M Zheng X Liu Y Li X Shan H. Evidence for Gastrointestinal Infection of SARS-Cov-2. Gastroenterology (2020) 158(6):1831–1833.e3. 10.1053/j.gastro.2020.02.055 32142773 PMC7130181

[19] Lee DB Huang E Ward HJ. Tight Junction Biology and Kidney Dysfunction. Am J Physiol Renal Physiol (2006) 290(1):F20–34. 10.1152/ajprenal.00052.2005 16339962

[20] Tian W Zhang N Jin R Feng Y Wang S Gao S. Immune Suppression in the Early Stage of COVID-19 Disease. Nat Commun (2020) 11(1):5859. 10.1038/s41467-020-19706-9 33203833 PMC7673112

[21] Wang W Uzzau S Goldblum SE Fasano A. Human Zonulin, a Potential Modulator of Intestinal Tight Junctions. J Cell Sci (2000) 113 Pt 24:4435–40. 10.1242/jcs.113.24.4435 11082037

[22] Goldblum SE Rai U Tripathi A Thakar M De Leo L Di Toro N. The Active Zot Domain (Aa 288-293) Increases ZO-1 and Myosin 1c Serine/Threonine Phosphorylation, Alters Interaction Between ZO-1 and Its Binding Partners, and Induces Tight Junction Disassembly Through Proteinase Activated Receptor 2 Activation. FASEB J (2011) 25(1):144–58. 10.1096/fj.10-158972 PMC3005425 20852064

[23] Fasano A Not T Wang W Uzzau S Berti I Tommasini A. Zonulin, a Newly Discovered Modulator of Intestinal Permeability, and Its Expression in Coeliac Disease. Lancet (2000) 355(9214):1518–9. 10.1016/S0140-6736(00)02169-3 10801176

[24] Rittirsch D Flierl MA Nadeau BA Day DE Huber-Lang MS Grailer JJ. Zonulin as Prehaptoglobin2 Regulates Lung Permeability and Activates the Complement System. Am J Physiol Lung Cell Mol Physiol (2013) 304(12):L863–72. 10.1152/ajplung.00196.2012 PMC3680747 23564505

[25] Skardelly M Armbruster FP Meixensberger J Hilbig H. Expression of Zonulin, C-Kit, and Glial Fibrillary Acidic Protein in Human Gliomas. Transl Oncol (2009) 2(3):117–20. 10.1593/tlo.09115 PMC2730142 19701495

[26] Lu R Wang W Uzzau S Vigorito R Zielke HR Fasano A. Affinity Purification and Partial Characterization of the Zonulin/Zonula Occludens Toxin (Zot) Receptor From Human Brain. J Neurochem (2000) 74(1):320–6. 10.1046/j.1471-4159.2000.0740320.x 10617135

[27] Di Micco S Musella S Scala MC Sala M Campiglia P Bifulco G. Analysis Revealed Potential Anti-SARS-Cov-2 Main Protease Activity by the Zonulin Inhibitor Larazotide Acetate. Front Chem (2020) 8:628609. 10.3389/fchem.2020.628609 33520943 PMC7843458

[28] Bourouiba L Dehandschoewercker E Bush JWM. Violent Expiratory Events: on Coughing and Sneezing. J Fluid Mech (2014) 745:537–63. 10.1017/jfm.2014.88

[29] Hoffmann M Kleine-Weber H Schroeder S Krüger N Herrler T Erichsen S. Sars-Cov-2 Cell Entry Depends on ACE2 and TMPRSS2 and is Blocked by a Clinically Proven Protease Inhibitor. Cell (2020) 181(2):271–280.e8. 10.1016/j.cell.2020.02.052 32142651 PMC7102627

[30] Kumar A Faiq MA Pareek V Raza K Narayan RK Prasoon P. Relevance of SARS-Cov-2 Related Factors ACE2 and TMPRSS2 Expressions in Gastrointestinal Tissue With Pathogenesis of Digestive Symptoms, Diabetes-Associated Mortality, and Disease Recurrence in COVID-19 Patients. Med Hypotheses (2020) 144:110271–1. 10.1016/j.mehy.2020.110271 PMC7487155 33254575

[31] Hamming I Timens W Bulthuis ML Lely AT Navis G van Goor H. Tissue Distribution of ACE2 Protein, the Functional Receptor for SARS Coronavirus A First Step in Understanding Sars Pathogenesis. J Pathol (2004) 203(2):631–7. 10.1002/path.1570 PMC7167720 15141377

[32] Lee JJ Kopetz S Vilar E Shen JP Chen K Maitra A. Relative Abundance of SARS-Cov-2 Entry Genes in the Enterocytes of the Lower Gastrointestinal Tract. Genes (Basel) (2020) 11(6):645–53. 10.3390/genes11060645 PMC7349178 32545271

[33] Holshue ML DeBolt C Lindquist S Lofy KH Wiesman J Bruce H. First Case of 2019 Novel Coronavirus in the United States. N Engl J Med (2020) 382(10):929–36. 10.1056/NEJMoa2001191 PMC7092802 32004427

[34] Wu Y Guo C Tang L Hong Z Zhou J Dong X. Prolonged Presence of SARS-Cov-2 Viral RNA in Faecal Samples. Lancet Gastroenterol Hepatol (2020) 5(5):434–5. 10.1016/S2468-1253(20)30083-2 PMC7158584 32199469

[35] Ye Q Wang B Zhang T Xu J Shang S. The Mechanism and Treatment of Gastrointestinal Symptoms in Patients With Covid-19. Am J Physiol Gastrointest Liver Physiol (2020) 319(2):G245–52. 10.1152/ajpgi.00148.2020 PMC7414235 32639848

[36] Cheung KS Hung IFN Chan PPY Lung KC Tso E Liu R. Gastrointestinal Manifestations of SARS-Cov-2 Infection and Virus Load in Fecal Samples From a Hong Kong Cohort: Systematic Review and Meta-Analysis. Gastroenterology (2020) 159(1):81–95. 10.1053/j.gastro.2020.03.065 32251668 PMC7194936

[37] Hindson J. Covid-19: Faecal-Oral Transmission? Nat Rev Gastroenterol Hepatol (2020) 17(5):259–9. 10.1038/s41575-020-0295-7 PMC7095230 32214231

[38] Qian Q Fan L Liu W Li J Yue J Wang M. Direct Evidence of Active Sars-Cov-2 Replication in the Intestine. Clin Infect Dis (2020) ciaa925:1–23. 10.1093/cid/ciaa925 32638022 PMC7454471

[39] Jin X Lian J-S Hu J-H Gao J Zheng L Zhang Y-M. Epidemiological, Clinical and Virological Characteristics of 74 Cases of Coronavirus-Infected Disease 2019 (Covid-19) With Gastrointestinal Symptoms. Gut (2020) 69(6):1002–9. 10.1136/gutjnl-2020-320926 PMC7133387 32213556

[40] Stanifer ML Kee C Cortese M Zumaran CM Triana S Mukenhirn M. Critical Role of Type Iii Interferon in Controlling Sars-Cov-2 Infection in Human Intestinal Epithelial Cells. Cell Rep (2020) 32(1):107863. 10.1016/j.celrep.2020.107863 32610043 PMC7303637

[41] Zang R Gomez Castro MF McCune BT Zeng Q Rothlauf PW Sonnek NM. TMPRSS2 and TMPRSS4 Promote SARS-Cov-2 Infection of Human Small Intestinal Enterocytes. Sci Immunol (2020) 5(47):1–14. 10.1126/sciimmunol.abc3582 PMC7285829 32404436

[42] Lamers MM Beumer J. van der Vaart J Knoops K Puschhof J Breugem TI. Sars-Cov-2 Productively Infects Human Gut Enterocytes. Science (2020) 369(6499):50–4. 10.1126/science.abc1669 PMC7199907 32358202

[43] Zhou J Li C Liu X Chiu MC Zhao X Wang D. Infection of Bat and Human Intestinal Organoids by SARS-Cov-2. Nat Med (2020) 26(7):1077–83. 10.1038/s41591-020-0912-6 32405028

[44] Guo Y Luo R Wang Y Deng P Song T Zhang M. Sars-Cov-2 Induced Intestinal Responses With a Biomimetic Human Gut-on-Chip. Sci Bull (Beijing) (2020) 66(8):783–93. 10.1016/j.scib.2020.11.015 PMC7704334 33282445

[45] Chin AWH Chu JTS Perera MRA Hui KPY Yen HL Chan MCW. Stability of SARS-Cov-2 in Different Environmental Conditions. Lancet Microbe (2020) 1(1):e10. 10.1016/S2666-5247(20)30003-3 32835322 PMC7214863

[46] Kopel J Perisetti A Gajendran M Boregowda U Goyal H. Clinical Insights Into the Gastrointestinal Manifestations of COVID-19. Dig Dis Sci (2020) 65(7):1932–9. 10.1007/s10620-020-06362-8 PMC7245177 32447742

[47] Hirose R Nakaya T Naito Y Daidoji T Watanabe Y Yasuda H. Mechanism of Human Influenza Virus Rna Persistence and Virion Survival in Feces: Mucus Protects Virions From Acid and Digestive Juices. J Infect Dis (2017) 216(1):105–9. 10.1093/infdis/jix224 28498998

[48] Weiss SR Navas-Martin S. Coronavirus Pathogenesis and the Emerging Pathogen Severe Acute Respiratory Syndrome Coronavirus. Microbiol Mol Biol Rev MMBR (2005) 69(4):635–64. 10.1128/MMBR.69.4.635-664.2005 PMC1306801 16339739

[49] Wan Y Shang J Graham R Baric RS Li F. Receptor Recognition by the Novel Coronavirus From Wuhan: An Analysis Based on Decade-Long Structural Studies of SARS Coronavirus. J Virol (2020) 94(7):1–9. 10.1128/JVI.00127-20 PMC7081895 31996437

[50] Akira S Takeda K. Toll-Like Receptor Signalling. Nat Rev Immunol (2004) 4(7):499–511. 10.1038/nri1391 15229469

[51] Tulic MK Hurrelbrink RJ Prêle CM Laing IA Upham JW Le Souef P. Tlr4 Polymorphisms Mediate Impaired Responses to Respiratory Syncytial Virus and Lipopolysaccharide. J Immunol (2007) 179(1):132–40. 10.4049/jimmunol.179.1.132 17579031

[52] Triantafilou K Triantafilou M. Coxsackievirus B4-Induced Cytokine Production in Pancreatic Cells is Mediated Through Toll-Like Receptor 4. J Virol (2004) 78(20):11313–20. 10.1128/JVI.78.20.11313-11320.2004 PMC521802 15452251

[53] Burzyn D Rassa JC Kim D Nepomnaschy I Ross SR Piazzon I. Toll-Like Receptor 4-Dependent Activation of Dendritic Cells by a Retrovirus. J Virol (2004) 78(2):576–84. 10.1128/jvi.78.2.576-584.2004 PMC368791 14694089

[54] Lammers KM Lu R Brownley J Lu B Gerard C Thomas K. Gliadin Induces an Increase in Intestinal Permeability and Zonulin Release by Binding to the Chemokine Receptor Cxcr3. Gastroenterology (2008) 135(1):194–204.e3. 10.1053/j.gastro.2008.03.023 18485912 PMC2653457

[55] Mukherjee S Karmakar S Babu SP. TLR2 and TLR4 Mediated Host Immune Responses in Major Infectious Diseases: A Review. Braz J Infect Dis (2016) 20(2):193–204. 10.1016/j.bjid.2015.10.011 26775799 PMC9427569

[56] Choudhury A Mukherjee S. In Silico Studies on the Comparative Characterization of the Interactions of SARS-Cov-2 Spike Glycoprotein With ACE-2 Receptor Homologs and Human Tlrs. J Med Virol (2020) 92(10):2105–13. 10.1002/jmv.25987 PMC7267663 32383269

[57] Sohn KM Lee S-G Kim HJ Cheon S Jeong H Lee J. Covid-19 Patients Upregulate Toll-Like Receptor 4-Mediated Inflammatory Signaling That Mimics Bacterial Sepsis. J Korean Med Sci (2020) 35(38):1–17. 10.3346/jkms.2020.35.e343 PMC7521960 32989935

[58] Tripathi A Lammers KM Goldblum S Shea-Donohue T Netzel-Arnett S Buzza MS. Identification of Human Zonulin, a Physiological Modulator of Tight Junctions, as Prehaptoglobin-2. Proc Natl Acad Sci USA (2009) 106(39):16799–804. 10.1073/pnas.0906773106 PMC2744629 19805376

[59] Ossovskaya VS Bunnett NW. Protease-Activated Receptors: Contribution to Physiology and Disease. Physiol Rev (2004) 84(2):579–621. 10.1152/physrev.00028.2003 15044683

[60] Noorbakhsh F Tsutsui S Vergnolle N Boven LA Shariat N Vodjgani M. Proteinase-Activated Receptor 2 Modulates Neuroinflammation in Experimental Autoimmune Encephalomyelitis and Multiple Sclerosis. J Exp Med (2006) 203(2):425–35. 10.1084/jem.20052148 PMC2118197 16476770

[61] Kelso EB Lockhart JC Hembrough T Dunning L Plevin R Hollenberg MD. Therapeutic Promise of Proteinase-Activated Receptor-2 Antagonism in Joint Inflammation. J Pharmacol Exp Ther (2006) 316(3):1017–24. 10.1124/jpet.105.093807 16260582

[62] Rallabhandi P Nhu QM Toshchakov VY Piao W Medvedev AE Hollenberg MD. Analysis of Proteinase-Activated Receptor 2 and TLR4 Signal Transduction: A Novel Paradigm for Receptor Cooperativity. J Biol Chem (2008) 283(36):24314–25. 10.1074/jbc.M804800200 PMC2528983 18622013

[63] Chi L Li Y Stehno-Bittel L Gao J Morrison DC Stechschulte DJ. Interleukin-6 Production by Endothelial Cells Via Stimulation of Protease-Activated Receptors is Amplified by Endotoxin and Tumor Necrosis Factor-Alpha. J Interferon Cytokine Res (2001) 21(4):231–40. 10.1089/107999001750169871 11359654

[64] Hamilton JR Frauman AG Cocks TM. Increased Expression of Protease-Activated Receptor-2 (PAR2) and PAR4 in Human Coronary Artery by Inflammatory Stimuli Unveils Endothelium-Dependent Relaxations to PAR2 and PAR4 Agonists. Circ Res (2001) 89(1):92–8. 10.1161/hh1301.092661 11440983

[65] Liu T Zhang J Yang Y Ma H Li Z Cheng J. The Role of Interleukin-6 in Monitoring Severe Case of Coronavirus Disease 2019. EMBO Mol Med (2020) 12(7):e12421. 10.15252/emmm.202012421 32428990 PMC7280589

[66] Gorham J Moreau A Corazza F Peluso L Ponthieux F Talamonti M. Interleukine-6 in Critically Ill COVID-19 Patients: A Retrospective Analysis. PloS One (2020) 15(12):e0244628. 10.1371/journal.pone.0244628 33382773 PMC7774924

[67] Oliviero S Cortese R. The Human Haptoglobin Gene Promoter: Interleukin-6-Responsive Elements Interact With a DNA-Binding Protein Induced by Interleukin-6. EMBO J (1989) 8(4):1145–51. 10.1002/j.1460-2075.1989.tb03485.x PMC400927 2787245

[68] Noris M Benigni A Remuzzi G. The Case of Complement Activation in COVID-19 Multiorgan Impact. Kidney Int (2020) 98(2):314–22. 10.1016/j.kint.2020.05.013 PMC7246017 32461141

[69] Risitano AM Mastellos DC Huber-Lang M Yancopoulou D Garlanda C Ciceri F. Complement as a Target in COVID-19? Nat Rev Immunol (2020) 20(6):343–4. 10.1038/s41577-020-0320-7 PMC7187144 32327719

[70] Desforges M Le Coupanec A Dubeau P Bourgouin A Lajoie L Dubé M. Human Coronaviruses and Other Respiratory Viruses: Underestimated Opportunistic Pathogens of the Central Nervous System? Viruses (2019) 12(1):1–28. 10.3390/v12010014 PMC7020001 31861926

[71] Esposito G Pesce M Seguella L Sanseverino W Lu J Sarnelli G. Can the Enteric Nervous System be an Alternative Entrance Door in SARS-Cov2 Neuroinvasion? Brain Behav Immun (2020) 87:93–4. 10.1016/j.bbi.2020.04.060 PMC7179488 32335192

[72] Buscarinu MC Fornasiero A Romano S Ferraldeschi M Mechelli R Reniè R. The Contribution of Gut Barrier Changes to Multiple Sclerosis Pathophysiology. Front Immunol (2019) 10:1916. 10.3389/fimmu.2019.01916 31555257 PMC6724505

[73] Mehta P McAuley DF Brown M Sanchez E Tattersall RS Manson JJ. Covid-19: Consider Cytokine Storm Syndromes and Immunosuppression. Lancet (Lond Engl) (2020) 395(10229):1033–4. 10.1016/S0140-6736(20)30628-0 PMC7270045 32192578

[74] Monteil V Kwon H Prado P Hagelkrüys A Wimmer RA Stahl M. Inhibition of SARS-Cov-2 Infections in Engineered Human Tissues Using Clinical-Grade Soluble Human Ace2. Cell (2020) 181(4):905–13.e7. 10.1016/j.cell.2020.04.004 32333836 PMC7181998

[75] Paniz-Mondolfi A Bryce C Grimes Z Gordon RE Reidy J Lednicky J. Central Nervous System Involvement by Severe Acute Respiratory Syndrome Coronavirus-2 (Sars-Cov-2). J Med Virol (2020) 92(7):699–702. 10.1002/jmv.25915 32314810 PMC7264598

[76] Idris F Muharram SH Zaini Z Alonso S Diah S. Invasion of a Murine in Vitro Blood-Brain Barrier Co-Culture Model by Dengue Virus Serotypes 1 to 4. Arch Virol (2019) 164(4):1069–83. 10.1007/s00705-019-04175-3 30783772

[77] Robinson CP Busl KM. Neurologic Manifestations of Severe Respiratory Viral Contagions. Crit Care Explor (2020) 2(4):e0107. 10.1097/CCE.0000000000000107 32426749 PMC7188429

[78] Daneman R Rescigno M. The Gut Immune Barrier and the Blood-Brain Barrier: Are They So Different? Immunity (2009) 31(5):722–35. 10.1016/j.immuni.2009.09.012 19836264

[79] Zlokovic BV. The Blood-Brain Barrier in Health and Chronic Neurodegenerative Disorders. Neuron (2008) 57(2):178–201. 10.1016/j.neuron.2008.01.003 18215617

[80] Sweeney MD Zhao Z Montagne A Nelson AR Zlokovic BV. Blood-Brain Barrier: From Physiology to Disease and Back. Physiol Rev (2019) 99(1):21–78. 10.1152/physrev.00050.2017 30280653 PMC6335099

[81] Radzikowska U Ding M Tan G Zhakparov D Peng Y Wawrzyniak P. Distribution of ACE2, Cd147, CD26, and Other Sars-Cov-2 Associated Molecules in Tissues and Immune Cells in Health and in Asthma, Copd, Obesity, Hypertension, and COVID-19 Risk Factors. Allergy (2020) 75(11):2829–45. 10.1111/all.14429 PMC7300910 32496587

[82] Fantini J Di Scala C Chahinian H Yahi N. Structural and Molecular Modelling Studies Reveal a New Mechanism of Action of Chloroquine and Hydroxychloroquine Against SARS-Cov-2 Infection. Int J Antimicrob Agents (2020) 55(5):105960. 10.1016/j.ijantimicag.2020.105960 32251731 PMC7128678

[83] Bocsik A Walter FR Gyebrovszki A Fülöp L Blasig I Dabrowski S. Reversible Opening of Intercellular Junctions of Intestinal Epithelial and Brain Endothelial Cells With Tight Junction Modulator Peptides. J Pharm Sci (2016) 105(2):754–65. 10.1016/j.xphs.2015.11.018 26869428

[84] Jacob A Alexander JJ. Complement and Blood-Brain Barrier Integrity. Mol Immunol (2014) 61(2):149–52. 10.1016/j.molimm.2014.06.039 25041699

[85] Widera D Martínez Aguilar R Cottrell GS. Toll-Like Receptor 4 and Protease-Activated Receptor 2 in Physiology and Pathophysiology of the Nervous System: More Than Just Receptor Cooperation? Neural Regener Res (2019) 14(7):1196–201. 10.4103/1673-5374.251290 PMC6425834 30804245

[86] Karyekar CS Fasano A Raje S Lu R Dowling TC Eddington ND. Zonula Occludens Toxin Increases the Permeability of Molecular Weight Markers and Chemotherapeutic Agents Across the Bovine Brain Microvessel Endothelial Cells. J Pharm Sci (2003) 92(2):414–23. 10.1002/jps.10310 12532391

[87] Silva AC Lobo JMS. Cytokines and Growth Factors. Adv Biochem Eng Biotechnol (2020) 171:87–113. 10.1007/10_2019_105 31384960

[88] Worldometers coronavirus: Available at: https://www.worldometers.info/coronavirus/?utm_campaign=homeAdUOA?Si (Accessed January 29, 2021).

